# Activation of Both TLR and NOD Signaling Confers Host Innate Immunity-Mediated Protection Against Microbial Infection

**DOI:** 10.3389/fimmu.2018.03082

**Published:** 2019-01-14

**Authors:** Huiting Zhou, Andrew P. Coveney, Ming Wu, Jie Huang, Siobhan Blankson, He Zhao, D. Peter O'Leary, Zhenjiang Bai, Yiping Li, H. Paul Redmond, Jiang Huai Wang, Jian Wang

**Affiliations:** ^1^Institute of Pediatric Research, Children's Hospital of Soochow University, Suzhou, China; ^2^Department of Academic Surgery, University College Cork, Cork University Hospital, Cork, Ireland; ^3^Department of Pediatric Surgery, Children's Hospital of Soochow University, Suzhou, China

**Keywords:** TLR signaling, NOD signaling, inflammatory response, antimicrobial activity, NF-κB pathway, phagosome maturation, microbial infection

## Abstract

The detection of microbial pathogens relies on the recognition of highly conserved microbial structures by the membrane sensor Toll-like receptors (TLRs) and cytosolic sensor NOD-like receptors (NLRs). Upon detection, these sensors trigger innate immune responses to eradicate the invaded microbial pathogens. However, it is unclear whether TLR and NOD signaling are both critical for innate immunity to initiate inflammatory and antimicrobial responses against microbial infection. Here we report that activation of both TLR and NOD signaling resulted in an augmented inflammatory response and the crosstalk between TLR and NOD led to an amplified downstream NF-κB activation with increased nuclear transactivation of p65 at both TNF-α and IL-6 promoters. Furthermore, co-stimulation of macrophages with TLR and NOD agonists maximized antimicrobial activity with accelerated phagosome maturation. Importantly, administration of both TLR and NOD agonists protected mice against polymicrobial sepsis-associated lethality with increased serum levels of inflammatory cytokines and accelerated clearance of bacteria from the circulation and visceral organs. These results demonstrate that activation of both TLR and NOD signaling synergizes to induce efficient inflammatory and antimicrobial responses, thus conferring protection against microbial infection.

## Introduction

Innate immunity constitutes the primary defense system of host against invading microbial pathogens (1). This mechanism of host defense involves the recognition of pathogens by germline-encoded host innate immune receptors or pattern-recognition receptors (PRRs) and in essence results in activation of the innate immune system (2, 3). Innate phagocytes including monocytes/macrophages and polymorphonuclear neutrophils (PMNs) express a variety of PRRs and thus are capable of detecting highly conserved and distinct molecular structures of pathogenic microorganisms, named pathogen-associated molecular patterns (PAMPs) as well as damage-associated molecular patterns (DAMPs) produced in the event of cellular and/or tissue injury (2, 4). Typical examples of PAMPs are lipopolysaccharide (LPS), bacterial lipoprotein (BLP), lipoteichoic acid (LTA), peptidoglycan (PGN), flagellin, and microbial nucleic acid. Detection of PAMPs by PRRs leads to the activation of intracellular signal transduction pathways, which in turn results in the initiation of inflammatory response and antimicrobial activity, culminating in the elimination of the invaded microbial pathogens (1, 3).

The two principal classes of PRRs predominantly involved in the recognition of molecular structures unique to microbial pathogens are Toll-like receptors (TLRs) and the nucleotide-binding oligomerization domain (NOD)-like receptors (NLRs) (3, 5). The transmembrane TLRs are the best-known PRRs, which possesses an extracellular domain responsible for the recognition of bacterial ligands at the cell surface or within endosomes. In particular, TLR4 is the primary receptor for LPS, thereby conferring the recognition of gram-negative bacteria (6, 7), whereas the heterodimmer of TLR2 with either TLR1 or TLR6 binds BLP and LTA, thus being accountable for gram-positive bacteria detection (8–10). On the other hand, the NLRs with up to 20 family members are located in the intracellular cytoplasm, thus serving as the cytosolic PRRs (5, 11). Among the NLR family members, NOD1 and NOD2 have been demonstrated to be capable of detecting different structural core patterns generated from PGN present in both gram-positive and gram-negative bacteria (11, 12). NOD1 senses PGN-derived peptides characterized by γ-D-glutamyl-meso-diaminopimelic acid (iE-DAP) from all gram-negative and certain gram-positive bacteria (13, 14), whereas NOD2 detects muramyl-dipeptide (MDP), a highly conserved PGN motif present in almost all bacteria (15, 16). NOD1 and NOD2 share similar tripartite structures, consisting of a C-terminal domain with the leucine-rich repeat (LRR) for microbial motif detection and ligand binding, a central nucleotide-binding domain (NBD) for ligand-induced self-oligomerization, and an N-terminal caspase-recruitment domain (CARD) for intracellular signal transduction (17–19). Upon bacterial ligand recognition and binding, both NOD1 and NOD2 recruit the adaptor protein, receptor-interacting protein 2 (RIP2), via homophilic CARD-CARD interactions, thereby activating downstream signal transduction pathways of nuclear factor-κB (NF-κB) and mitogen-activated protein kinase (MAPK), thus initiating transcription of the targeted genes and consequently necessitating the production of inflammatory cytokines and chemokines, antimicrobial peptides, and type I interferons (IFNs) (12, 20).

There is growing evidence for the importance of NOD1 and NOD2 by functioning as intracellular PPRs to sense the fragments of bacterial PGN in host innate immunity-associated protection against microbial infection. Consequently, NOD1-deficient mice display an increased vulnerability to a number of microbial pathogens including *Helicobacter pylori, Streptococcus pneumoniae*, and *Clostridium* difficile (20–22), whereas mice with deficiency in NOD2 were more susceptible to infections caused by *Streptococcus pneumoniae, Citrobacter rodentium*, and *Staphylococcus aureus* (23–25). This enhanced susceptibility to microbial infection observed in NOD1- and NOD2-deficient mice correlated closely to the diminished recruitment of PMNs and impaired antimicrobial activity of macrophages (21–24). Furthermore, both NOD1 and NOD2 are implicated in the development of certain inflammatory diseases, as supported by the association of LRR mutations in NOD2 with an increased risk for developing Crohn's disease (26) and a complex insertion-deletion polymorphism in NOD1 with early onset of inflammatory bowel disease (27). More recently, NOD1 and NOD2 have been implicated to participate in PGN-independent inflammatory responses by not only sensing of virus and parasite infections but also monitoring of Rho GTPpase activation and endoplasmic reticulum stress to maintain the homeostasis of intracellular environments (28).

It was generally presumed that the membrane-bound TLR2 and TLR4 survey the extracellular bacteria, whereas the cytosolic NOD1 and NOD2 detect the intracellular microbial pathogens (5). Indeed, NOD1 and NOD2 play a critical role in protection against the intracellular *Legionella pneumophila*-induced pneumonia by promoting PMN recruitment into the lung (29), whereas deficiency in either NOD1 or NOD2 led to an impaired bacterial clearance of *Chlamydophila pneumophia*, a gram-negative intracellular pathogen (30). However, emerging evidence has revealed that quite a number of extracellular bacteria such as *Staphylococus aureus* and *Streptococus pneumonia* can also be detected by NOD1 and NOD2 (21, 23, 25, 31, 32). Conversely, TLR2 and TLR4 are implicated in the recognition of both extracellular and intracellular bacteria (6, 9, 10, 33, 34). Of note, it is demonstrated that the induction and acceleration of phagosome maturation upon microbial infection occurs via a TLR-dependent manner where TLR signaling tightly controls phagosome maturation and the destruction of engulfed microbial pathogens in the case of extracellular bacteria as well as intracellular bacteria (35). Therefore, it is more likely that extracellular TLR2/TLR4 and intracellular NOD1/NOD2 work in synergy to necessitate an efficient and vigorous innate immune response for eradication of a variety of the invaded microbial pathogens. In the present study, we identified that TLR and NOD signaling are both critical for innate immunity to trigger a strong inflammatory response and to initiate an efficient antimicrobial activity, thereby facilitating host defense to combat against microbial infection.

## Materials and Methods

### Reagents and Antibodies

The TLR2 agonist BLP, a synthetic bacterial lipopeptide (Pam3Cys-Ser-Lys4-OH), and the TLR4 agonist ultrapure LPS from *E. coli* serotype O55:B5 were purchased from EMC Microcollections (Tubingen, Germany) and InvivoGen (San Diego, CA), respectively. The NOD1 agonist L-Ala-γ-D-Glu-mDAP (Tri-DAP) and NOD2 agonist MDP were obtained from InvivoGen. Antibodies (Abs) that recognize TLR4, NOD1, NOR2, myeloid differentiation factor 88 (MyD88), IL-1 receptor-associated kinase-1 (IRAK-1), CARD9, and RIP2 were purchased from Cell Signaling Technology (Beverly, MA), Santa Cruz Biotechnology (Santa Cruz, CA), and Abcam (Cambridge, MA), respectively. Abs that recognize NF-κB p65, phosphor-p65 at Ser586, the inhibitor of κBα (IκBα), phosphor-IκBα at Ser32/36, MAPK p38, and phospho-p38 at Th180/Try182 were purchased from Cell Signaling Technology. All culture medium and reagents for cell cultures were obtained from Invitrogen Life Technologies (Paisley, Scotland, U.K.). All other chemicals, unless indicated, were purchased from Sigma-Aldrich (St. Louis, MO).

### Mice, Murine Macrophage Isolation and Cultures

Pyrogen-free, 8- to 10-week-old C3H/HeN mice, TLR2- and TLR4-deficient mice on the C3H background, C57BL/6 mice, NOD1- and NOD2-deficient mice on the C57BL/6 background were obtained from Harlan (Oxon, UK), Jackson Laboratories (Bar Harbor, ME), and Carsten J. Kirschning at Technische Universitat Munchen, Munich, Germany. Mice were housed in barrier cages under controlled environmental conditions (12/12 h light/dark cycle, 55 ± 5% humidity, 23°C) and had free access to standard laboratory chow and water. All animal studies were conducted with the ethical approval granted from the Institutional Animal Care and Use Committee of Soochow University and the Ethics Committee of University College Cork, and complied with the animal welfare act. The methods applied in the present study were performed in accordance with the approved guidelines.

Peritoneal macrophages were collected from wild-type, TLR4- and TLR2-deficient, and NOD1- and NOD2-deficient mice by peritoneal lavage and incubated with DMEM containing 10% heat-inactivated fetal calf serum (FCS) in 24-well plates (Falcon, Lincoln Park, NJ) for 90 min to remove non-adherent cells as previously described (36, 37). Bone marrow-derived macrophages (BMMs) were isolated from the femurs and tibias of wild-type, TLR4- and TLR2-deficient, and NOD1- and NOD2-deficient mice, and cultured in DMEM containing 20% heat-inactivated FCS, penicillin (100 units/ml), streptomycin sulfate (100 μg/ml), and supplemented with 10 ng/ml of recombinant mouse macrophage colony-stimulating factor (CSF) (R&D Systems, Minneapolis, MN) for 7 days at 37°C in a humidified 5% CO2 atmosphere as previously described (36, 37). The purity of both peritoneal macrophages and BMMs was >95%, as confirmed by FACScan analysis of the positive F4/80 antigen (Ag) staining with a rat anti-mouse F4/80 Ab (Serotec, Oxford, U.K.).

Isolated peritoneal macrophages or BMMs were incubated with PBS, LPS (10 ng/ml), BLP (10 ng/ml), Tri-DAP (5 μg/ml), MDP (5 μg/ml), LPS + Tri-DAP (10 ng/ml + 5 μg/ml), LPS + MDP (10 ng/ml + 5 μg/ml), BLP + Tri-DAP (10 ng/ml + 5 μg/ml), and BLP + MDP (10 ng/ml + 5 μg/ml) for 6 h, and further challenged with gram-positive or gram-negative bacteria to assess their ability in bacterial phagocytosis, killing, and phagosome maturation.

### Cytokine Measurement

Isolated peritoneal macrophages or BMMs were plated onto 96-well plates (Falcon) at 2 × 10^4^ cells/well and incubated with PBS as the control or stimulated with LPS (10 ng/ml), BLP (10 ng/ml), Tri-DAP (5 μg/ml), MDP (5 μg/ml), and their combinations for 12 h. Cell-free supernatants were collected and stored at −80°C until analysis. Concentrations of inflammatory cytokines TNF-α, IL-6, IL-12p70, and chemokine CXCL2 in the supernatant were assessed by cytometric bead array (BD Biosciences, San Joes, CA) and ELISA (R&D Systems), respectively.

### FACScan Analysis for Phagocytic Receptor Expression

Isolated peritoneal macrophages were incubated with PBS, LPS (10 ng/ml), BLP (10 ng/ml), Tri-DAP (5 μg/ml), MDP (5 μg/ml), and their combinations for 2 h, and stained with anti-complement receptor type 3 (CR3) (BD PharMingen, San Diego, CA) and anti-FcγIII/II receptor (FcγR) (BD PharMingen) monoclonal antibodies (mAbs) conjugated with PE or FITC. PE- or FITC-conjugated isotype-matched mAbs (BD PharMingen) were used as the control. FACScan analysis was performed from at least 10,000 events for detecting the surface expression of CR3 and FcγR on macrophages using CellQuest software (BD Biosciences).

### Bacteria and Bacterial Uptake, Ingestion, and Killing

Gram-positive *Staphylococcus aureus* (*S. aureus*) and gram-negative *Salmonella typhimurium* (*S. typhimurium*) were obtained from American Type Culture Collection (ATCC, Manassas, VA) and the National University of Ireland Culture Collection, respectively. Bacteria were cultured at 37°C in trypticase soy broth (Merck, Darmstadt, Germany), harvested at the mid-logarithmic growth phase, washed twice, and resuspended in PBS for *in vitro* use. The concentration of resuspended bacteria was determined and adjusted spectrophotometrically at 550 nm.

Bacterial uptake, phagocytosis, and intracellular bacterial killing were determined as previously described (38, 39). Briefly, *S. aureus* and *S. typhimurium* were heat-killed at 95°C for 20 min and labeled with 0.1% FITC (Sigma-Aldrich). After stimulation with LPS, BLP, Tri-DAP, MDP, and their combinations for 6 h as mentioned above, isolated peritoneal macrophages or BMMs were incubated with heat-killed, FITC-labeled *S. aureus* or *S. typhimurium* at a ratio of 1/20 (macrophage/bacteria) at 37°C for 30 min. Bacterial uptake was assessed by FACScan analysis and bacterial ingestion was further determined after the external fluorescence of the bound, but non-ingested, bacteria was quenched with 0.025% crystal violet (Sigma-Aldrich). Intracellular bacterial killing was assessed by incubation of macrophages with live *S. aureus* or *S. typhimurium* (macrophage/bacteria = 1/20) at 37°C for 60 min in the presence or absence of cytochalasin B (5 μg/ml) (Sigma-Aldrich). After macrophages were lysed, total, and extracellular bacterial killing were determined by incubation of serial 10-fold dilutions of the lysates on tryptone soy agar (Merck) plates at 37°C for 24 h. Intracellular bacterial killing was calculated according to the total and extracellular bacterial killing.

### Measurement of Phagosomal pH

Phagosome luminal pH was assessed as previously described (40, 41). Briefly, heat-killed *S. aureus* and *S. typhimurium* were doubly labeled with 5 μg/ml carboxyfluorescein-SE (a pH-sensitive fluorescent probe) (Molecular Probes, Eugene, OR) and 10 μg/ml carboxytetramethylrhodamine-SE (a pH-insensitive fluorescent probe) (Molecular Probes). After stimulation with LPS, BLP, Tri-DAP, MDP, and their combinations for 6 h, isolated peritoneal macrophages or BMMs were pulsed with the labeled bacteria (macrophage/bacteria = 1/20) for 20 min, and then chased at 37°C for the indicated time periods. Macrophage-based mean fluorescence intensity (MFI) of fluorescein on FL1 and rhodamine on FL2 were simultaneously detected by FACScan analysis using CellQuest software (BD Biosciences). Phagosomal pH was calculated according to the ratio of fluorescein/rhodamine fluorescence using a calibration curve.

### Assessment of Phagosome Maturation in a Cell-Free Organelle System

After stimulation with LPS, BLP, Tri-DAP, MDP, and their combinations for 6 h, isolated peritoneal macrophages or BMMs were labeled with a red fluorescent cell membrane linker PKH26 (20 μM) (Sigma-Aldrich) for subsequent phagosome recognition, as previously described (40, 42). PKH26-labeled macrophages were pulsed and chased with heat-killed *S. aureus* or *S. typhimurium* (macrophage/bacteria = 1/20) at 37°C for the indicated time periods. Cells were lysed in a hypotonic buffer, and phagosomes were prepared by centrifugation. The isolated phagosomes were permeabilised with 0.2% saponin (Sigma-Aldrich) and stained with FITC-conjugated anti-LAMP-1 mAb (Abcam) that specially recognizes late endosomes/lysosomes, or FITC-conjugated isotype-matched mAb (Abcam) as the control. The green MFI of LAMP-1 on the positive red fluorescent events (phagosomes that have ingested bacteria), representing phagolysosome fusion and/or phagosome maturation, was quantitatively assessed by FACScan analysis using CellQuest software (BD Biosciences).

### FACScan Analysis for Phosphorylated NF-κB p65 and MAPK p38

Isolated BMMs were incubated with PBS, BLP (10 ng/ml), Tri-DAP (5 μg/ml), MDP (5 μg/ml), and their combinations for various time periods. For determination of intracellular NF-κB p65 and MAPK p38 phosphorylation, BMMs were fixed and permeabilized simultaneously with Phosflow fix and perm buffers (BD Biosciences) for 30 min on ice. Cells were then stained with anti-phospho p65 and anti-phospho p38 mAbs conjugated with PE or Alexa Fluor 488 (Cell Signaling Technology). PE or Alexa Fluor 488-conjugated isotype-matched mAbs (Cell Signaling Technology) were used as the control. FACScan analysis was performed from at least 10,000 events for detecting the intracellular staining of phosphorylated NF-κB p65 and MAPK p38 in BMMs using CellQuest software (BD Biosciences).

### Western Blot Analysis

Following stimulation of isolated BMMs with LPS (10 ng/ml), Tri-DAP (5 μg/ml), MDP (5 μg/ml), and their combinations for the indicated time periods, cells were collected, washed with ice-cold PBS, and lysed on ice in cell lysis buffer (Cell Signaling Technology), supplemented with 1 mM phenylmethylsulfonyl fluoride and protease inhibitor cocktail (Roche Life Science, Indianapolis, IN). The resultant lysates were centrifuged and supernatants containing the cytoplasmic proteins were collected. Protein concentrations were determined using a micro bicinchoninic acid (BCA) protein assay (Pierce, Rockford, IL). Equal amounts of protein extracts were separated on SDS-polyacrylamide gels and trans-blotted onto polyvinylidene difluoride (PVDF) membranes (Schleicher and Schuell, Dassel, Germany). The membrane was blocked for 1 h at room temperature with PBS containing 0.05% Tween-20 and 5% nonfat milk, and probed overnight at 4°C with the respected primary Abs. Blots were then incubated with appropriate horseradish peroxidase-conjugated secondary Abs (Dako, Cambridge, U.K.) at room temperature for 1 h, developed with SuperSignal chemiluminescent substrate (Pierce), and captured with LAS-3000 imaging system (Fujifilm, Tokyo, Japan).

### Chromatin Immunoprecipitation (ChIP) Assay

ChIP assay was performed using the ChIP-IT Express kit (Active Motif, Carlsbad, CA) according to the manufacturer's instructions. Briefly, isolated BMMs were stimulated with LPS (10 ng/ml), BLP (10 ng/ml), Tri-DAP (5 μg/ml), MDP (5 μg/ml), and their combinations for 1 h, and then washed with PBS and fixed with 1% formaldehyde at room temperature for 10 min. The cells were lysed in ice-cold lysis buffer and sheared by sonication to generate 200–1,000 bp DNA fragments. Immunoprecipitation was carried out by incubation of the diluted sonicates with protein G magnetic beads and a specific anti-NF-κB p65 Ab (Santa Cruz Biotechnology) with rotation overnight. Protein G magnetic beads were collected with magnetic stand (Active Motif) and washed extensively. Protein–DNA complexes were eluted, the cross-link was reversed, and proteins were digested with proteinase K. Immunoprecipitated DNA and nonimmunoprecipitated DNA (input control) were amplified by quantitative PCR (qPCR) using the following promoter-specific primers: mouse TNF-α (sense-5′-TCCTTGATGCCTGGGTGTCCC-3′ and antisense-5′-GCAGACGGCCGCCTTTATAGC-3′) and mouse IL-6 (sense-5′-TCCAATCAGCCCCACCCACTC-3′ and antisense-5′-GGTGGGCTCCAGAGCAGAATG-3′).

### Cecal Ligation and Puncture (CLP)-Induced Polymicrobial Sepsis

Pyrogen-free, 8- to 10-week-old male C3H/HeN mice received intraperitoneal injection of 200 μl PBS, LPS (1 mg/kg), BLP (1 mg/kg), Tri-DAP (15 mg/kg), LPS + Tri-DAP (1 mg/kg + 15 mg/kg), and BLP + Tri-DAP (1 mg/kg + 15 mg/kg), respectively, 6 h before septic challenge. Polymicrobial sepsis was induced using a CLP method as previously described (40, 43). Briefly, mice were anesthetized by intramuscular injection of 150 μl of a ketamine/xylazine admixture (150 μl ketamine + 150 μl xylazine made up to 1 ml with 0.9% saline). A midline laparotomy was performed at which the cecum was delivered, ligated at the base, just distal to the ileocaecal juncture with a 2/0 mersilk tie. A single through puncture was then made distal to the ligature with a 17G needle. The cecum was returned to the peritoneal cavity and the abdomen was closed with 6/0 prolene sutures. Survival rates were recorded and monitored for at least 7 days. Blood samples were collected at 2 and 6 h post CLP, and serum TNF-α and IL-6 were assessed by cytometric bead array (BD Biosciences).

### Enumeration of Bacteria in the Blood and Visceral Organs

Bacterial counts were determined as previously described (39, 40). Briefly, mice were culled at 12 and 24 h post CLP-induced polymicrobial sepsis. Blood samples were obtained by retinal artery puncture, and the dissected liver and spleen were homogenized in sterile PBS. Serial 10-fold dilutions of heparinized whole blood and organ homogenates in sterile water containing 0.5% Triton X-100 (Sigma-Aldrich) were plated on brain heart infusion agar (BD Biosciences) and incubated for 24 h at 37°C for determination of bacterial colony-forming unit (CFU).

### Statistical Analysis

All data are expressed as the mean ± SD. Statistical analysis was performed using the log rank test for survival, and the ANOVA or Mann-Whitney *U* test for all others with GraphPad software version 5.01 (Prism, La Jolla, CA). Differences were judged to be statistically significant when the *p*-value was less than 0.05.

## Results

### Activation of TLR and NOD Signaling Is Both Required to Induce a Strong Inflammatory Response

**Figure 1:**
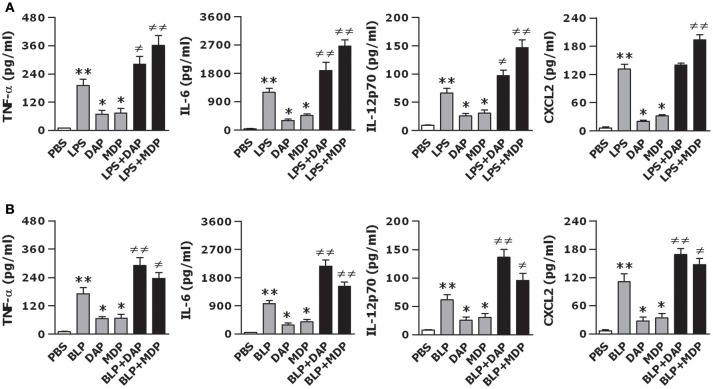
A combined stimulation of LPS or BLP with Tri-DAP or MDP results in augmented inflammatory cytokine and chemokine release. Peritoneal macrophages and BMMs isolated from wild-type mice were stimulated with LPS (10 ng/ml), Tri-DAP (5 μg/ml), MDP (5 μg/ml), and their combinations **(A)** or BLP (10 ng/ml), Tri-DAP (5 μg/ml), MDP (5 μg/ml), and their combinations **(B)** for 12 h. Macrophages incubated with PBS were used as the control. TNF-α, IL-6, IL-12p70, and CXCL2 concentrations in the supernatants were assessed by cytometric bead array. Data are expressed as mean ± SD from five to six independent experiments in duplicate. *p < 0.05, **p < 0.01 vs. macrophages incubated with PBS; ^≠^*p* < 0.05, ^≠≠^*p* < 0.01 vs. macrophages stimulated with LPS, BLP, Tri-DAP, or MDP alone.

We first stimulated macrophages isolated from wild-type mice with the TLR4 agonist LPS, NOD1 agonist Tri-DAP, NOD2 agonist MDP, and their combinations for 12 h to assess the production of inflammatory cytokines and chemokines. As shown in Figure 1A, stimulation of macrophages with LPS led to an increased release of inflammatory cytokines TNF-α, IL-6, IL-12p70, and chemokine CXCL2 (*p* < 0.01 vs. PBS-treated macrophages), whereas Tri-DAP or MDP stimulation caused moderate but significant increases in TNF-α, IL-6, IL-12p70, and CXCL2 release (*p* < 0.05 vs. PBS-treated macrophages). Of note, a combined stimulation of LPS with Tri-DAP or MDP maximized the inflammatory response with substantially augmented release of TNF-α, IL-6, IL-12p70, and CXCL2 when compared to the response observed with LPS, Tri-DAP, or MDP alone (*p* < 0.05, *p* < 0.01). Consistent with the finding from the TLR4 agonist LPS stimulation, stimulation of macrophages with the TLR2 agonist BLP also induced a markedly increased release of TNF-α, IL-6, IL-12p70, and CXCL2 (*p* < 0.01 vs. PBS-treated macrophages), which was further augmented by a combination of BLP plus Tri-DAP or MDP (*p* < 0.05, *p* < 0.01 vs. macrophages stimulated with BLP, Tri-DAP, or MDP alone) (Figure 1B). These results indicate that activation of both TLR and NOD signaling in macrophages leads to an augmented inflammatory response.

**Figure 2:**
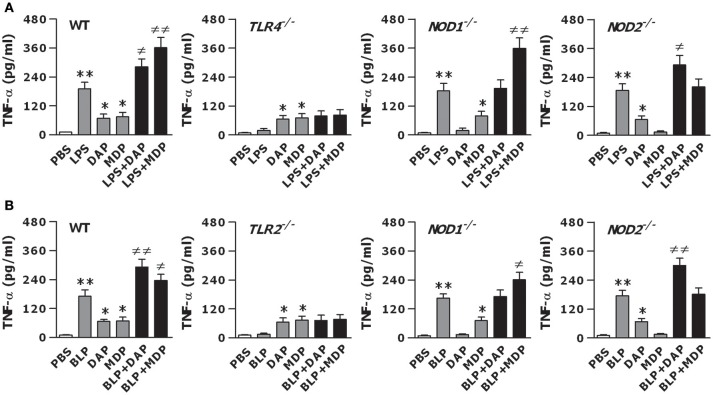
The augmented inflammatory response is dependent on the intact of both TLR and NOD signaling. Peritoneal macrophages and BMMs isolated from wild-type, TLR4- and TLR2-deficient, and NOD1- and NOD2-deficient mice were stimulated with LPS (10 ng/ml), Tri-DAP (5 μg/ml), MDP (5 μg/ml), and their combinations **(A)** or BLP (10 ng/ml), Tri-DAP (5 μg/ml), MDP (5 μg/ml), and their combinations **(B)** for 12 h. Macrophages incubated with PBS were used as the control. TNF-α concentrations in the supernatants were assessed by cytometric bead array. Data are expressed as mean ± SD from five to six independent experiments in duplicate. *p < 0.05, **p < 0.01 vs. macrophages incubated with PBS; ^≠^*p* < 0.05, ^≠≠^*p* < 0.01 vs. macrophages stimulated with LPS, BLP, Tri-DAP, or MDP alone.

To examine whether TLR and NOD signaling are both critical for the observed optimal inflammatory response, we further stimulated macrophages isolated from TLR4- and TLR2-deficient, and NOD1- and NOD2-deficient mice with LPS, BLP, Tri-DAP, MDP, and their combinations. LPS stimulation failed to induce TNF-α release and was unable to augment Tri-DAP- or MDP-induced TNF-α release in TLR4-deficinet macrophages (Figure 2A). Moreover, the amplified TNF-α release induced by a combined stimulation of LPS with the NOD1 agonist Tri-DAP or NOD2 agonist MDP observed in wild-type macrophages was receded in NOD1- and NOD2-deficient macrophages, respectively (Figure 2A). Similarly, BLP also lost its stimulatory ability in TLR2-deficient macrophages, and failed to augment Tri-DAP- or MDP-induced TNF-α release in NOD1- or NOD2-deficient macrophages (Figure 2B). These results suggest that the augmented inflammatory response by a combined stimulation of TLR and NOD agonists is entirely dependent on intact TLR and NOD signaling.

### The Crosstalk Between TLR and NOD Signaling Leads to Augmented Activation of NF-κB and Nuclear Transactivation of P65 at Both TNF-α and IL-6 Promoters

**Figure 3:**
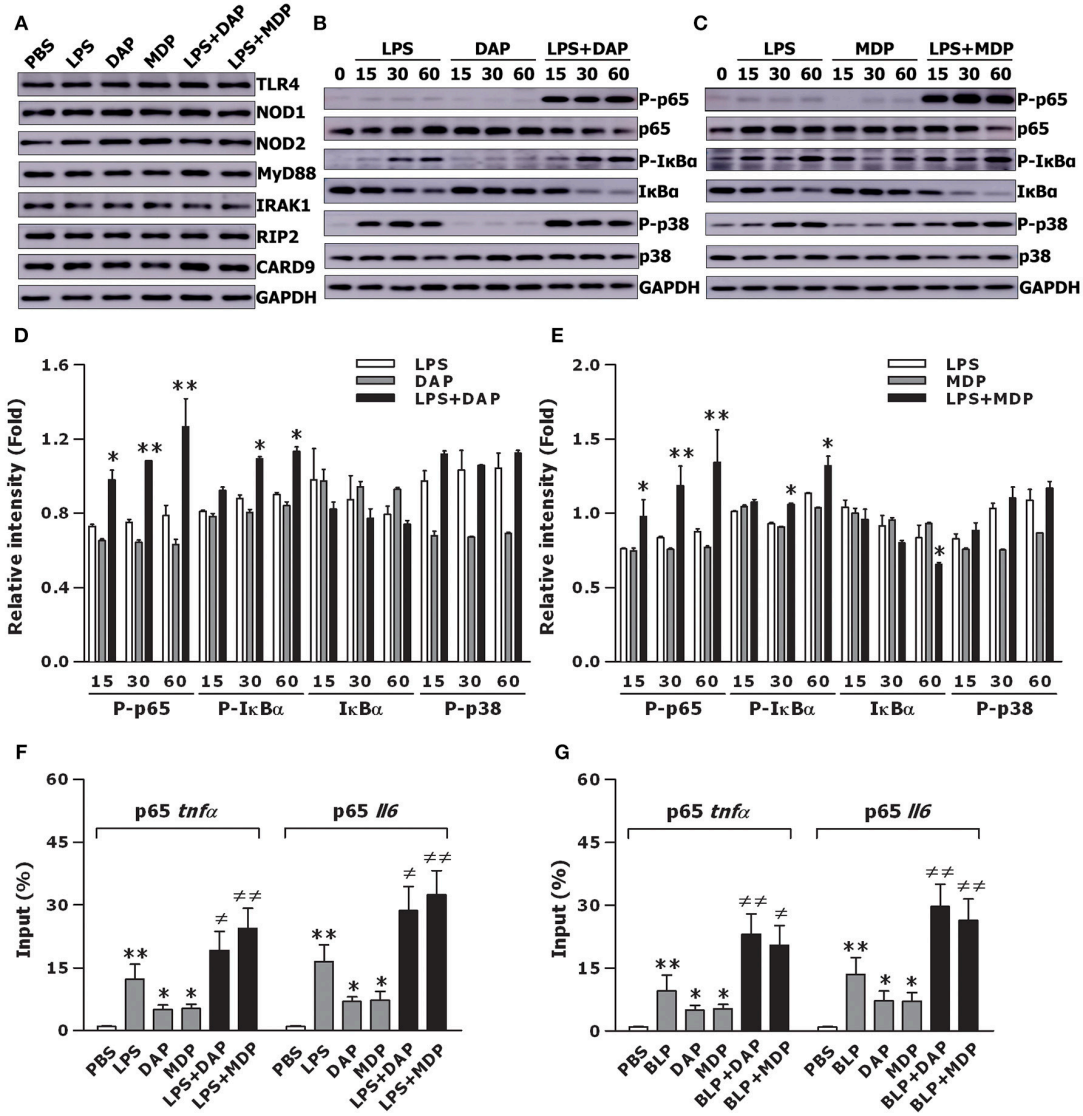
Stimulation of macrophages with the combined TLR and NOD agonists amplifies downstream NF-κB activation and augments NF-κB p65 binding to TNF-α and IL-6 promoters. Isolated BMMs were stimulated with LPS (10 ng/ml), Tri-DAP (5 μg/ml), MDP (5 μg/ml), and their combinations either for 30 min **(A)** or for the indicated time periods **(B,C)**. Cytoplasmic proteins were extracted and subjected to immunoblotting for detection of either TLR4, NOD1, NOD2, MyD88, IRAK1, RIP2, and CARD9 **(A)** or total and phosphorylated p65 (P-p65), total and phosphorylated IκBα (P- IκBα), and total and phosphorylated p38 (P-p38) **(B,C)**. Results shown represent one experiment from a total of three to four separate experiments. The intensity of P-65, total IκBα, P-IκBα, and P-p38 signal in each band was normalized by GAPDH **(D,E)**. Data are expressed as mean ± SD from three to four separate experiments. *p < 0.05, **p < 0.01 vs. macrophages stimulated with LPS, Tri-DAP, or MDP alone. Isolated BMMs were stimulated with LPS (10 ng/ml), Tri-DAP (5 μg/ml), MDP (5 μg/ml), and their combinations **(F)** or BLP (10 ng/ml), Tri-DAP (5 μg/ml), MDP (5 μg/ml), and their combinations **(G)** for 1 h. The binding of NF-κB p65 to TNF-α and IL-6 promoters was assessed by ChIP analysis and expressed as percentage of input. Data are expressed as mean ± SD from four independent experiments in duplicate. *p < 0.05, **p < 0.01 vs. macrophages incubated with PBS; ^≠^*p* < 0.05, ^≠≠^*p* < 0.01 vs. macrophages stimulated with LPS, BLP, Tri-DAP, or MDP alone.

To examine whether the crosstalk between TLR and NOD signaling results in an enhanced activation of intracellular signal transduction pathways, thus causing an augmented inflammatory response, we assessed TLR- and NOD-mediated upstream and downstream pathways in macrophages stimulated with TLR and NOD agonists. Stimulation of BMMs with LPS, Tri-DAP, MDP, and their combinations did not affect TLR- and NOD-mediated upstream pathways including the expression of TLR4, NOD1, NOD2, MyD88, IRAK1, RIP2, and CARD9 (Figure 3A). However, stimulation of BMMs with a combination of LPS plus Tri-DAP resulted in a vigorous activation in the downstream NF-κB pathway with considerably increased phosphorylation of NF-κB p65 at Ser586 and IκBα at Ser32/36 (*p* < 0.05, *p* < 0.01), but had no augmentative effect on phosphorylation of MAPK p38 at Th180/Try182, when compared to BMMs stimulated with LPS or Tri-DAP alone (Figures 3B,D). A markedly enhanced expression of phosphorylated NF-κB p65 at Ser586 and IκBα at Ser32/36 was also observed in BMMs stimulated by LPS in combination with MDP (*p* < 0.05, *p* < 0.01 vs. BMMs stimulated with LPS or MDP alone) (Figures 3C,E). We also stimulated BMMs with BLP, Tri-DAP, MDP, and their combinations, and observed a substantially augmented activation of NF-κB p65 in BMMs stimulated with a combination of BLP plus Tri-DAP (*p* < 0.01) (Figure S1A) or MDP (*p* < 0.05) (Figure S1B) compared with BMMs stimulated with BLP, Tri-DAP, or MDP alone. Again, stimulation of BMMs by BLP in combination with Tri-DAP (Figure S1C) or MDP (Figure S1D) did not induce a further activation of MAPK p38 compared with BMMs stimulated with BLP, Tri-DAP, or MDP alone. Consistent with the amplified downstream NF-κB activation by a combined stimulation of TLR and NOD agonists, stimulation of BMMs with a combination of LPS plus Tri-DAP or MDP strongly augmented the nuclear transactivation of NF-κB p65 at both TNF-α and IL-6 promoters (*p* < 0.05, *p* < 0.01 vs. BMMs stimulated with LPS, Tri-DAP, or MDP alone) (Figure 3F). A substantially enhanced recruitment of NF-κB p65 to either the TNF-α promoter or IL-6 promoter was also observed in BMMs stimulated by BLP in combination with Tri-DAP or MDP (*p* < 0.05, *p* < 0.01 vs. BMMs stimulated with BLP, Tri-DAP, or MDP alone) (Figure 3G). These results indicate that the crosstalk between TLR and NOD signaling by a co-stimulation with TLR and NOD agonists triggers an amplified downstream activation of the NF-κB pathway with subsequently augmented nuclear transactivation of p65 at both TNF-α and IL-6 promoters.

### Activation of TLR and NOD Signaling Is Both Essential for an Efficient Innate Phagocyte-Associated Bactericidal Activity

**Figure 4:**
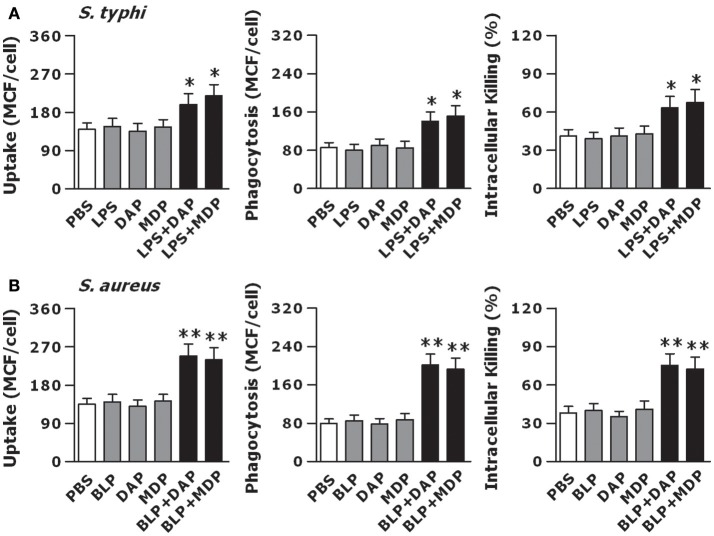
Stimulation of macrophages with the combined TLR and NOD agonists maximizes the innate phagocyte-associated antimicrobial activity. Peritoneal macrophages isolated from wild-type mice were stimulated with LPS (10 ng/ml), Tri-DAP (5 μg/ml), MDP (5 μg/ml), and their combinations **(A)** or BLP (10 ng/ml), Tri-DAP (5 μg/ml), MDP (5 μg/ml), and their combinations **(B)** for 6 h, and further incubated with either FITC-conjugated *S. typhimurium* (*S. typhi*) **(A)**, FITC-conjugated *S. aureus* **(B)** for 30 min to assess bacterial uptake and phagocytosis or live *S. typhi* **(A)**, live *S. aureus* **(B)** for 60 min to assess intracellular bacterial killing. Bacterial uptake and phagocytosis were expressed as mean channel fluorescence (MCF) per cell. Data are expressed as mean ± SD from four to five independent experiments in triplicate. *p < 0.05, **p < 0.01 vs. macrophages stimulated with LPS, BLP, Tri-DAP, or MDP alone.

We stimulated macrophages isolated from wild-type mice with LPS, Tri-DAP, MDP, and their combinations for 6 h, and further challenged these macrophages with gram-negative *S. typhimurium* to assess bacterial uptake, phagocytosis, and intracellular killing. Stimulation with LPS, Tri-DAP, or MDP alone did not affect macrophage-associated bactericidal activity; however, a combined stimulation of LPS with Tri-DAP or MDP significantly enhanced uptake and phagocytosis of *S. typhimurium* with substantially increased intracellular killing of the engulfed *S. typhimurium* (*p* < 0.05 vs. macrophages stimulated with LPS, Tri-DAP, or MDP alone) (Figure 4A). BLP stimulation in combination with Tri-DAP or MDP also resulted in an augmented bactericidal activity against gram-positive *S. aureus*, as represented by significantly enhanced uptake, phagocytosis, and intracellular killing of *S. aureus* (*p* < 0.01 vs. macrophages stimulated with BLP, Tri-DAP, or MDP alone), whereas stimulation with BLP, Tri-DAP, or MDP alone had no such effect (Figure 4B). Nevertheless, a combined stimulation of LPS or BLP with either Tri-DAP or MDP significantly augmented macrophage-associated bactericidal activity against gram-positive *S. aureus* (*p* < 0.05 vs. macrophages stimulated with LPS, Tri-DAP, or MDP alone) (Figure S2A) and gram-negative *S. typhimurium* (*p* < 0.01 vs. macrophages stimulated with BLP, Tri-DAP, or MDP alone) (Figure S2B). These results indicate that activation of both TLR and NOD signaling in macrophages is required to induce an enhanced antimicrobial response.

**Figure 5:**
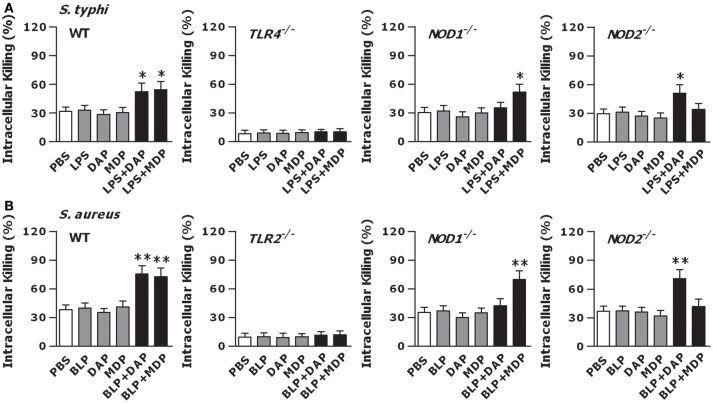
TLR and NOD signaling are both required for an efficient macrophage-mediated intracellular bacterial killing. Peritoneal macrophages isolated from wild-type, TLR4- and TLR2-deficient, and NOD1- and NOD2-deficient mice were stimulated with LPS (10 ng/ml), Tri-DAP (5 μg/ml), MDP (5 μg/ml), and their combinations **(A)** or BLP (10 ng/ml), Tri-DAP (5 μg/ml), MDP (5 μg/ml), and their combinations **(B)** for 6 h, and further incubated with live *S. typhimurium* (*S. typhi*) **(A)** or live *S. aureus* **(B)** for 60 min to assess intracellular bacterial killing. Data are expressed as mean ± SD from four to five independent experiments in triplicate. *p < 0.05, **p < 0.01 vs. macrophages stimulated with LPS, BLP, Tri-DAP, or MDP alone.

We next stimulated macrophages isolated from TLR4- and TLR2-deficient, and NOD1- and NOD2-deficient mice with LPS, BLP, Tri-DAP, MDP, and their combinations for 6 h, and further challenged these macrophages with live bacteria to assess the intracellular bacterial killing. The augmented bactericidal activity observed in wild-type macrophages following a combined stimulation of LPS with Tri-DAP or MDP was totally abolished in TLR4-deficient macrophages and selectively lost in NOD1- and NOD2-deficient macrophages upon live gram-negative *S. typhimurium* (Figure 5A) or gram-positive *S. aureus* (Figure S3A) infection. An enhanced intracellular killing of the engulfed *S. aureus* (Figure 5B) or *S. typhimurium* (Figure S3B) seen in wild-type macrophages after stimulation with a combination of BLP plus Tri-DAP or MDP was also absent in TLR2-deficient macrophages as well as in NOD1- or NOD2-deficient macrophages. These results suggest that TLR and NOD signaling are both critical for an efficient phagocyte-associated bactericidal activity.

### Activation of TLR and NOD Signaling Enhances Phagocytic Receptor Expression and Accelerates Phagosome Maturation

We first examined whether co-stimulation of macrophages with TLR and NOD agonists induces an enhanced expression of phagocytic receptors. Stimulation with LPS, but not Tri-DAP or MDP, increased surface expression of CR3 and FcγR on macrophages, and a combined stimulation of LPS with Tri-DAP or MDP led to a further upregulation of CR3 and FcγR expression (Figure S4A). An enhanced expression of CR3 and FcγR was also observed in macrophages stimulated with BLP alone, which was further augmented in macrophages stimulated by a combination of BLP plus Tri-DAP or MDP (Figure S4B). All phagocytic processes including the engulfment of microbial pathogens within the phagocyte are primarily driven by a tightly controlled rearrangement of the actin cytoskeleton or actin polymerization (44), we next assessed actin polymerization in macrophages stimulated with TLR and NOD agonists. Significantly enhanced actin polymerizations as represented by the F-actin ratio were observed in macrophages stimulated by LPS in combination with Tri-DAP or MDP, but not by LPS, Tri-DAP, or MDP alone, upon gram-positive *S. aureus* or gram-negative *S. typhimurium* infection (*p* < 0.05, *p* < 0.01 vs. macrophages stimulated with LPS, Tri-DAP, or MDP alone) (Figure S5A). A combined stimulation of BLP with Tri-DAP or MDP also caused markedly increases in actin polymerization in response to *S. aureus* or *S. typhimurium* infection (*p* < 0.01 vs. macrophages stimulated with BLP, Tri-DAP, or MDP alone) (Figure S5B).

**Figure 6:**
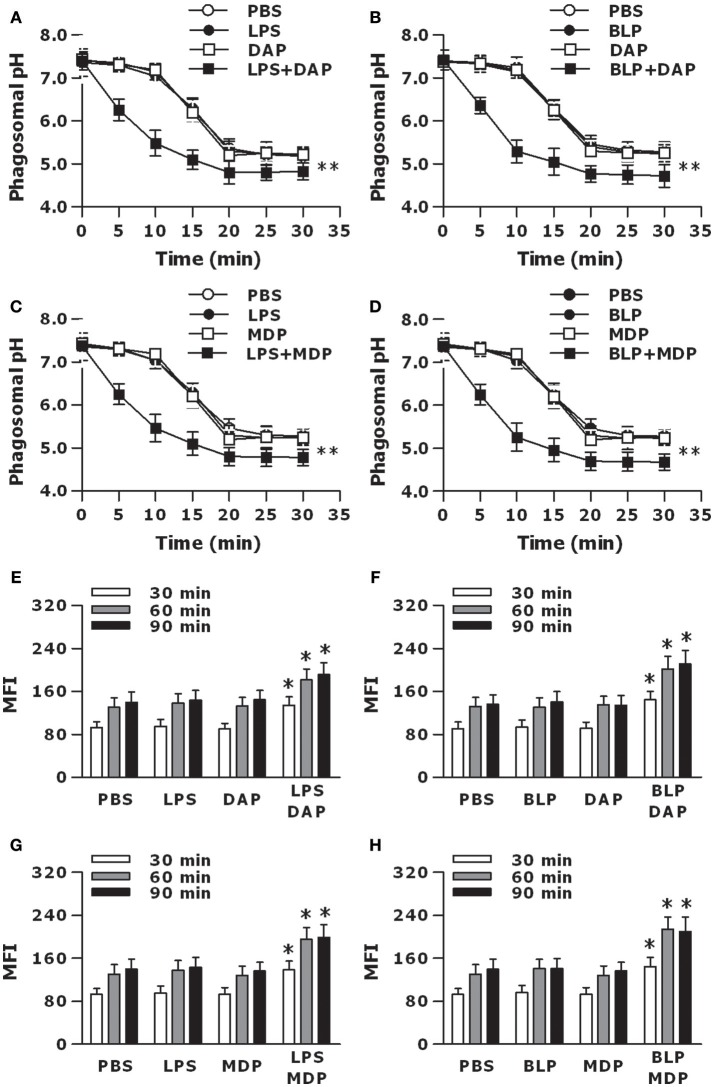
Stimulation of macrophages with the combined TLR and NOD agonists accelerates phagosome maturation. Peritoneal macrophages isolated from wild-type mice were stimulated with LPS (10 ng/ml), Tri-DAP (5 μg/ml), MDP (5 μg/ml), and their combinations **(A,C,E,G)** or BLP (10 ng/ml), Tri-DAP (5 μg/ml), MDP (5 μg/ml), and their combinations **(B,D,F,H)** for 6 h, and further incubated with heat-killed bacteria for the indicated time periods. **(A–D)**, Phagosomal pH was kinetically measured in peritoneal macrophages after being chased with fluorescent probe-coupled *S. typhimurium* **(A,C)** or *S. aureus* **(B,D)**. **(E–H)**, Phagolysosome fusion was assessed in phagosomes isolated from peritoneal macrophages after being chased with *S. typhimurium* **(E,G)** or *S. aureus* **(F,H)**, and expressed as mean fluorescence intensity (MFI). Data are expressed as mean ± SD from five to six independent experiments in duplicate. *p < 0.05, **p < 0.01 vs. macrophages stimulated with LPS, BLP, Tri-DAP, or MDP alone.

We further determined whether activation of TLR and NOD signaling by their specific agonists results in an accelerated phagosome maturation. Stimulation of macrophages with LPS, Tri-DAP, or MDP alone had no effect on phagosomal acidification; however, a combined stimulation of LPS with Tri-DAP or MDP substantially accelerated phagosomal acidification after engulfment of gram-negative *S. typhimurium* (*p* < 0.01 vs. macrophages stimulated with LPS, Tri-DAP, or MDP alone) (Figures 6A,C). A similar acceleration in phagosomal acidification was also seen in macrophages stimulated by a combination of BLP plus Tri-DAP or MDP after ingestion of gram-positive *S. aureus* (*p* < 0.01 vs. macrophages stimulated with BLP, Tri-DAP, or MDP alone) (Figures 6B,D). Consistent with an accelerated phagosomal acidification, macrophages stimulated by a combination of LPS or BLP plus either Tri-DAP or MDP displayed significantly increased phagolysosome fusion at 30, 60, and 90 min after ingestion of *S. typhimurium* (*p* < 0.05 vs. macrophages stimulated with LPS, Tri-DAP, or MDP alone) (Figures 6E,G) and *S. aureus* (*p* < 0.05 vs. macrophages stimulated with BLP, Tri-DAP, or MDP alone) (Figures 6F,H), as determined in a cell-free organelle system.

Upregulation and activation of membrane-trafficking regulators and lysosomal enzymes in macrophages during the process of bacterial phagocytosis are critical events for subsequent phagolysosome fusion and efficient killing of the ingested microbial pathogens (45, 46), we next examined whether co-stimulation of macrophages with TLR and NOD agonists upregulates membrane-trafficking regulator and lysosomal enzyme expression. Stimulation of macrophages with LPS, but not Tri-DAP or MDP, led to increased expression of membrane-trafficking regulators Rab10 and Stx1A as well as lysosomal enzymes Camp and Acp5 (*p* < 0.05 vs. PBS-treated macrophages); however, a combined stimulation of LPS with Tri-DAP or MDP maximized mRNA expressing levels of Rab10, Stx1A, Camp, and Acp5 when compared with macrophages stimulated with LPS, Tri-DAP, or MDP alone (*p* < 0.05, *p* < 0.01) (Figure S6A). A similar augmented expression of Rab10, Stx1A, Camp, and Acp5 was also observed in macrophages stimulated by BLP in combination with Tri-DAP or MDP (*p* < 0.01 vs. macrophages stimulated with LPS, Tri-DAP, or MDP alone) (Figure S6B). These results indicate that activation of both TLR and NOD signaling in macrophages results in upregulated phagocytic receptor expression, enhanced actin polymerization, accelerated phagosome maturation, and increased membrane-trafficking regulators and lysosomal enzymes in response to microbial infection.

### Activation of TLR and NOD Signaling Confers Protection Against Polymicrobial Sepsis With Enhanced Inflammatory Cytokines and Accelerated Bacterial Clearance

**Figure 7:**
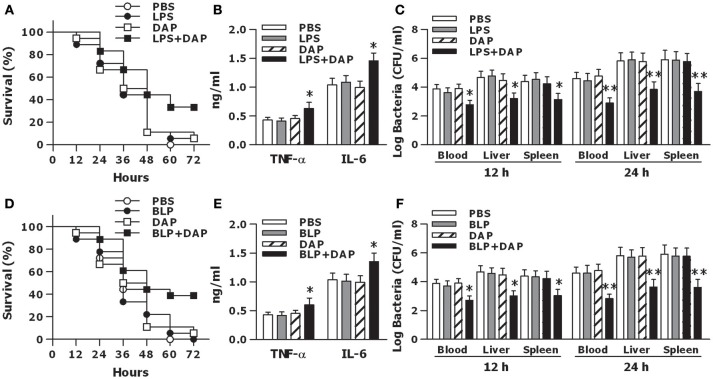
Activation of both TLR and NOD signaling protects mice against CLP-induced polymicrobial sepsis. C3H/HeN mice treated with PBS, LPS, Tri-DAP, and LPS + Tri-DAP **(A–C)**, or PBS, BLP, Tri-DAP, and BLP + Tri-DAP **(D–F)** for 6 h were subjected to CLP-induced polymicrobial sepsis. **(A,D)** Kaplan-Meier survival curve shows significantly improved survival in mice received LPS + Tri-DAP compared to mice received PBS, LPS, or Tri-DAP alone (*n* = 18 per group) **(A)** and in mice received BLP + Tri-DAP compared to mice received PBS, BLP, or Tri-DAP alone (*n* = 18 per group) **(D)** post septic challenge. **(B,E)** Data shown are the results of peak serum levels of TNF-α at 2 h and IL-6 at 6 h post septic challenge. **(C,F)**. Bacterial clearance from the blood and visceral organs collected at 12 and 24 h post septic challenge was expressed as log CFU/ml. Data in **(B,E)**, and **(C,F)** are mean ± SD of five to six mice per time point and representative of three separate experiments. *p < 0.05, **p < 0.01 vs. mice received LPS, BLP, or Tri-DAP alone.

To further clarify whether activation of both TLR and NOD signaling *in vivo* affords protection against microbial infection, wild-type mice were pretreated with PBS, LPS, Tri-DAP, or LPS plus Tri-DAP for 6 h, and further challenged with CLP-induced polymicrobial sepsis. Survival rates were recorded and monitored for at least 7 days. All mice receiving PBS succumbed within 60 h of septic challenge, while mice receiving LPS or Tri-DAP alone had a similar mortality rate at 95%, respectively, upon septic challenge (Figure 7A). However, mice that received a combination of LPS plus Tri-DAP were shown to be more resistant to polymicrobial sepsis, with an overall survival of 34% compared with the survival rate of 0% in mice that received PBS (*p* = 0.0112), 5% in mice that received LPS alone (*p* = 0.0275), and 5% in mice that received Tri-DAP alone (*p* = 0.0375) (Figure 7A). Consistent with a substantial survival advantage, mice treated with LPS plus Tri-DAP displayed moderate but significantly increased serum peak levels of TNF-α at 2 h and IL-6 at 6 h post septic challenge (*p* < 0.05 vs. mice treated with LPS or Tri-DAP alone) (Figure 7B). Furthermore, substantially reduced bacterial counts in the blood, spleen, and liver were observed at 12 and 24 h post septic challenge in mice treated with LPS plus Tri-DAP (*p* < 0.05, *p* < 0.01 vs. mice treated with LPS or Tri-DAP alone) (Figure 7C), indicating an accelerated bacterial clearance in these mice. Stimulation with a combination of BLP plus Tri-DAP also protected mice against polymicrobial sepsis-associated lethality, with a significant reduction in mortality from 100% in mice receiving PBS (*p* = 0.0091), 100% in mice receiving BLP alone (*p* = 0.0085), and 95% in mice receiving Tri-DAP alone (*p* = 0.0298) to 61% in mice receiving BLP plus Tri-DAP (Figure 7D). Consistent with the findings in mice treated with LPS plus Tri-DAP, mice treated with BLP plus Tri-DAP showed increased serum TNF-α and IL-6 (*p* < 0.05 vs. mice treated with BLP or Tri-DAP alone) (Figure 7E), and reduced bacterial load in the circulation and visceral organs (*p* < 0.05 vs. mice treated with BLP or Tri-DAP alone) (Figure 7F) post septic challenge. These results demonstrate that activation of both TLR and NOD signaling by the combined TLR and NOD agonists protects mice against microbial sepsis-associated lethality, which is associated with simultaneously augmented both inflammatory and antimicrobial responses.

## Discussion

The individual importance of the membrane-bound TLR2/4 and the cytosolic NOD1/2 during microbial infection has been well documented (6, 9, 22, 23, 29, 34); however, it is largely undefined whether TLR and NOD signaling are both critical for host defense to induce an efficient innate immune response, thereby facilitating the host to eradicate the invaded microbial pathogens. In the current study, we demonstrate that activation of both TLR and NOD signaling in macrophages by a combined stimulation of either the TLR2 agonist BLP or the TLR4 agonist LPS plus the NOD1 agonist Tri-DAP or the NOD2 agonist MDP augments not only the inflammatory response as represented by the upregulated downstream NF-κB activation and increased proinflammatory cytokine and chemokine release, but also the antimicrobial activity as represented by the accelerated phagosome maturation and enhanced bacterial killing. In line with our *in vitro* findings, activation of both TLR and NOD signaling by a combination of TLR and NOD agonists *in vivo* enhances serum proinflammatory cytokines and accelerates clearance of bacteria from the circulation and visceral organs, thus conferring protection against polymicrobial sepsis-associated lethality.

Proinflammatory cytokines and chemokines, serving as the major participants in innate immunity-initiated inflammatory response, are essential for the elimination of microbial pathogens from the body (1–3, 10, 12). We first examined whether activation of both TLR and NOD signaling induced a strong inflammatory response. In comparison with the response induced by LPS, BLP, Tri-DAP, or MDP alone, a combined stimulation of macrophages with LPS or BLP plus Tri-DAP or MDP maximized the inflammatory response with markedly enhanced release of proinflammatory cytokines TNF-α, IL-6, IL-12p70, and chemokine CXCL2. Our results are consistent with previous reports where co-stimulation of murine macrophages and/or human monocytes/dendritic cells with agonists of TLR2/3/4/5/9 and NOD1/2 augments the inflammatory response with synergistically increased production and release of inflammatory cytokines including TNF-α, IL-1β, IL-6, IL-8, and IL-12 (13, 47–50), and furthermore, LPS or LTA stimulation in combination with MDP led to an augmented release of TNF-α and IL-6 not only in naive macrophages but also in LPS- and LTA-tolerant macrophages (51). Notably, our results differ considerably from the previous findings, by revealing that deficiency in TLR (TLR2 or TLR4) and NOD (NOD1 or NOD2) predominantly receded the amplified TNF-α release characterized in wild-type macrophages in response to co-stimulation with TLR and NOD agonists, indicating that the augmented inflammatory response observed in the present study is entirely dependent on intact TLR and NOD signaling. We further examined whether the crosstalk between TLR and NOD by co-stimulation with their agonists led to an enhanced activation of TLR- and NOD-mediated signal transduction pathways. Although stimulation of BMMs by LPS or BLP in combination with Tri-DAP or MDP failed to activate the upstream pathways including TLR4, NOD1, NOD2, MyD88, IRAK1, RIP2, and CARD9, a vigorous activation of the downstream NF-κB pathway with markedly upregulated expression in phosphorylated IκBα and NF-κB p65 was observed in BMMs co-stimulated by LPS or BLP plus Tri-DAP or MDP. Moreover, a significantly enhanced nuclear transactivation of NF-κB p65 at both TNF-α and IL-6 promoters was evident in BMMs stimulated by the combination of TLR and NOD agonists. Thus, the crosstalk between TLR and NOD signaling triggers an augmented downstream NF-κB activation with increased recruitment of NF-κB p65 to both TNF-α and IL-6 promoters, which provides the mechanistic explanation for an amplified release of proinflammatory cytokines observed in macrophages co-stimulated with TLR and NOD agonists. It has been reported that in addition to an enhanced NF-κB activation, substantial activation of MAPKs including markedly upregulated phosphorylation of p38, ERK, and JNK was associated with the increased cytokine response in LPS-tolerant macrophages after re-stimulation with MDP or KF1B, a NOD1 agonist (51). However, we did not observe any further increase in phosphorylated MARK p38 in naive macrophages co-stimulated by LPS or BLP plus Tri-DAP or MDP.

Receptor-associated recognition of invading microbial pathogens initiates the antimicrobial response of host innate immunity, and subsequently professional phagocytes such as macrophages ingest these pathogens via phagocytic receptors and kill them within the phagocyte through a process of lysosome fusion with the pathogen-containing phagosomes (35, 46, 52). We first assessed whether macrophages co-stimulated with TLR and NOD agonists developed an augmented antimicrobial activity upon bacterial infection. Significantly increased uptake, phagocytosis, and intracellular killing of gram-positive *S. aureus* and gram-negative *S. typhimurium* were observed in macrophages co-stimulated with LPS or BLP plus Tri-DAP or MDP. Importantly, deficiency in either TLR2/4 or NOD1/2 dramatically impaired the intracellular killing of *S. aureus* and *S. typhimurium* in macrophages co-stimulated with TLR and NOD agonists, demonstrating that TLR and NOD signaling are both essential for an efficient phagocyte-associated bactericidal activity. The phagocytic receptors CR3 and FcγR contribute to phagocyte-related uptake, engulfment, and killing of invading microbial pathogens, whereas defects in CR3 and/or FcγR are associated with an impaired antimicrobial response (53–55). Moreover, the event of phagosome maturation which is typified by phagosomal acidification and phagosome/lysosome fusion following the engulfment of microbial pathogens by professional phagocytes is a critical step in the killing and degradation of the ingested pathogens within the phagocyte, and therefore plays a crucial role in innate immunity against microbial infection (46, 52). We next asked whether activation of TLR and NOD signaling results in enhanced phagocytic receptor expression and accelerates phagosome maturation, thus facilitating phagocytosis and killing of microbial pathogens. By measuring the surface expression of CR3 and FcγR as well as phagosomal acidification and phagolysosome fusion in macrophages stimulated by a combination of LPS or BLP with Tri-DAP or MDP, we confirmed significantly upregulated phagocytic receptor expression and accelerated phagosome maturation in these macrophages. We further revealed that stimulation of macrophages by LPS or BLP in combination with Tri-Dap or MDP strongly enhanced actin polymerization, an event crucial for phagocyte-associated engulfment of microbial pathogens (44), and substantially increased Rab10 and Stx1A, two membrane-trafficking regulators involved in promoting phagolysosome fusion (45), and Camp and Acp5, two lysosomal enzymes responsible for killing of the ingested microbial pathogens (46). Thus, we demonstrate for the first time that in addition to inducing an augmented inflammatory response, activation of both TLR and NOD signaling initiates an efficient antimicrobial activity characterized by substantially increased uptake, phagocytosis, and killing of the ingested bacteria via the upregulation phagocytic receptor expression, promotion of actin polymerization, acceleration of phagosome maturation, and enhancement of membrane-trafficking regulators and lysosomal enzymes.

The membrane-bound TLR2/4 and the cytosolic NOD1/2 play key roles in host innate defense-associated protection against microbial infection by sensing the presence of microbial pathogens including both extracellular and intracellular bacteria (29, 31, 33, 34), while deficiency in either TLR2/4 or NOD1/2 show an increased susceptibility to infections caused by a variety of bacteria (6, 9, 21, 23, 30). In the current study, we found that activation of TLR and NOD signaling are both required for innate immunity to induce a strong inflammatory response, and simultaneously, an efficient antimicrobial activity. The remaining question to be answered is whether activation of both TLR and NOD signaling *in vivo* confers protection against microbial sepsis. To address this, we treated mice with LPS, BLP, Tri-DAP, and their combinations and further challenged these mice with CLP-induced polymicrobial sepsis. We revealed that mice receiving LPS plus Tri-DAP or BLP plus Tri-DAP were more resistant to CLP-induced polymicrobial sepsis with significantly improved survival when compared to mice receiving LPS, BLP, and Tri-DAP alone. Moreover, this protection was closely associated with increased serum proinflammatory cytokines and accelerated bacterial clearance observed in mice receiving both TLR and NOD agonists. Together, we demonstrate in the present study that TLR and NOD signaling are both critical for host innate immunity to induce a strong inflammatory response, and simultaneously, efficient antimicrobial elimination. More importantly, activation of both TLR and NOD signaling *in vivo* confers the protection against polymicrobial sepsis-associated lethality via increased proinflammatory cytokines and accelerated bacterial clearance. These findings clearly identify that TLR and NOD signaling synergize to strengthen the innate immune response against microbial infection.

## Author Contributions

HuZ, APC, HPR, JHW, and JW: designed the study. HuZ, APC, MW, JH, SB, HeZ, ZB, and YL: performed experiments. HuZ, APC, MW, DPO, and JHW: analyzed data. HuZ, APC, HPR, JHW, and JW: wrote the manuscript, and all authors reviewed the manuscript.

### Conflict of Interest Statement

The authors declare that the research was conducted in the absence of any commercial or financial relationships that could be construed as a potential conflict of interest.
