# Supplementary material for: Activation of Both TLR and NOD Signaling Confers Host Innate Immunity-Mediated Protection Against Microbial Infection
Source: Front Immunol. 2019 Jan 14;9:3082. doi: 10.3389/fimmu.2018.03082 (PMC6339916; doi:10.3389/fimmu.2018.03082)
Supplement: Supplementary file 1 [file Data_Sheet_1.doc]

**Figure S1**


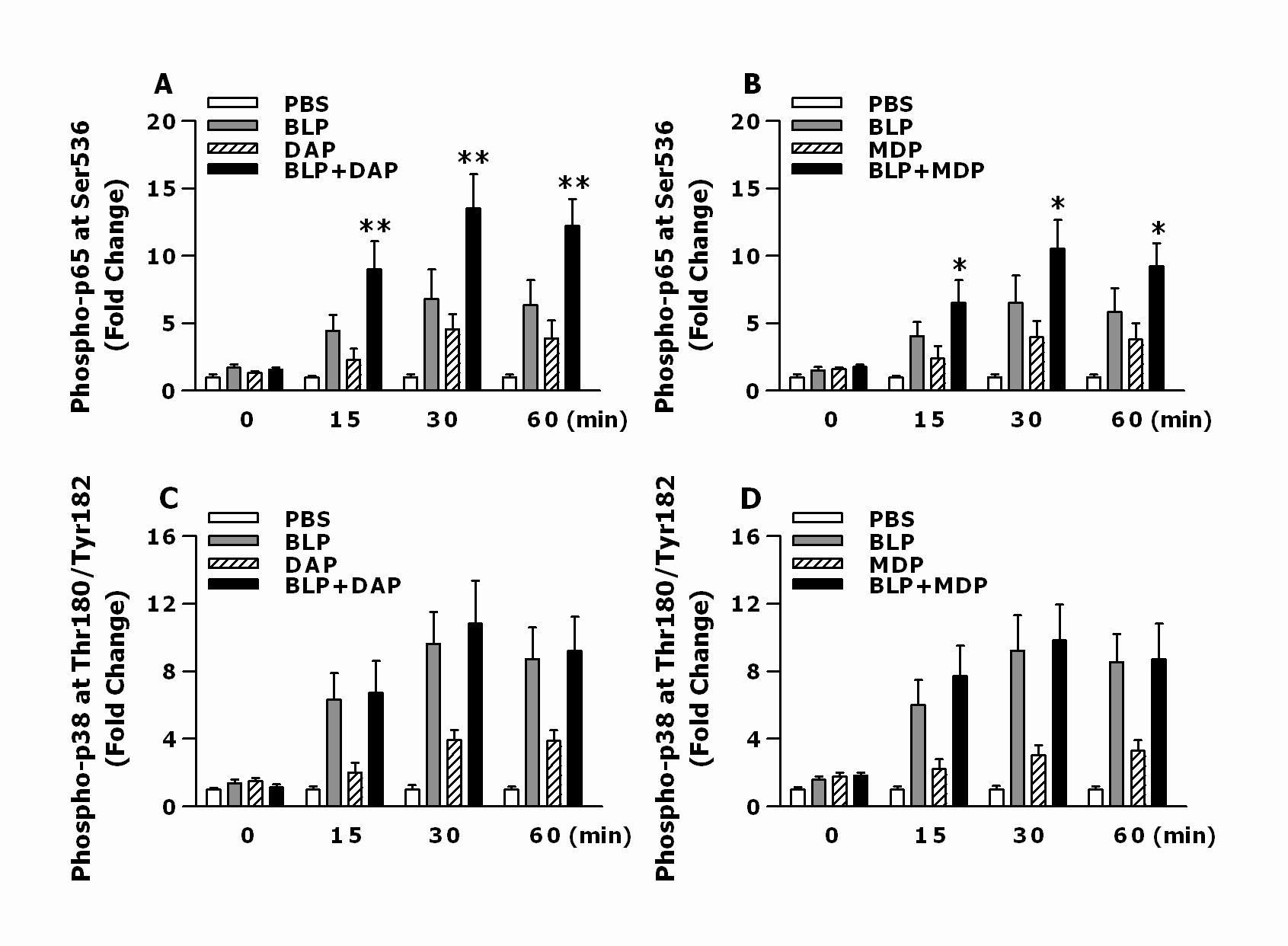


**Figure S1**. BLP stimulation in combination with Tri-DAP or MDP results in an amplified downstream activation of the NF-κB pathway. BMMs isolated from wild-type mice were stimulated with BLP (10 ng/ml), Tri-DAP (5 µg/ml), MDP (5 µg/ml), and their combinations for the indicated time periods. The intracellular phosphorylation of NF-κB at Ser536 (**A**, **B**) and MAPK p38 at Thr180/Tyr182 (**C**, **D**) was assessed by FACScan analysis and expressed as fold change of the mean fluorescence intensity (MFI). Data are expressed as mean ± SD from five independent experiments in duplicate. **p*<0.05, ***p*<0.01 versus macrophages stimulated with BLP, Tri-DAP, or MDP alone.

**Figure S2**


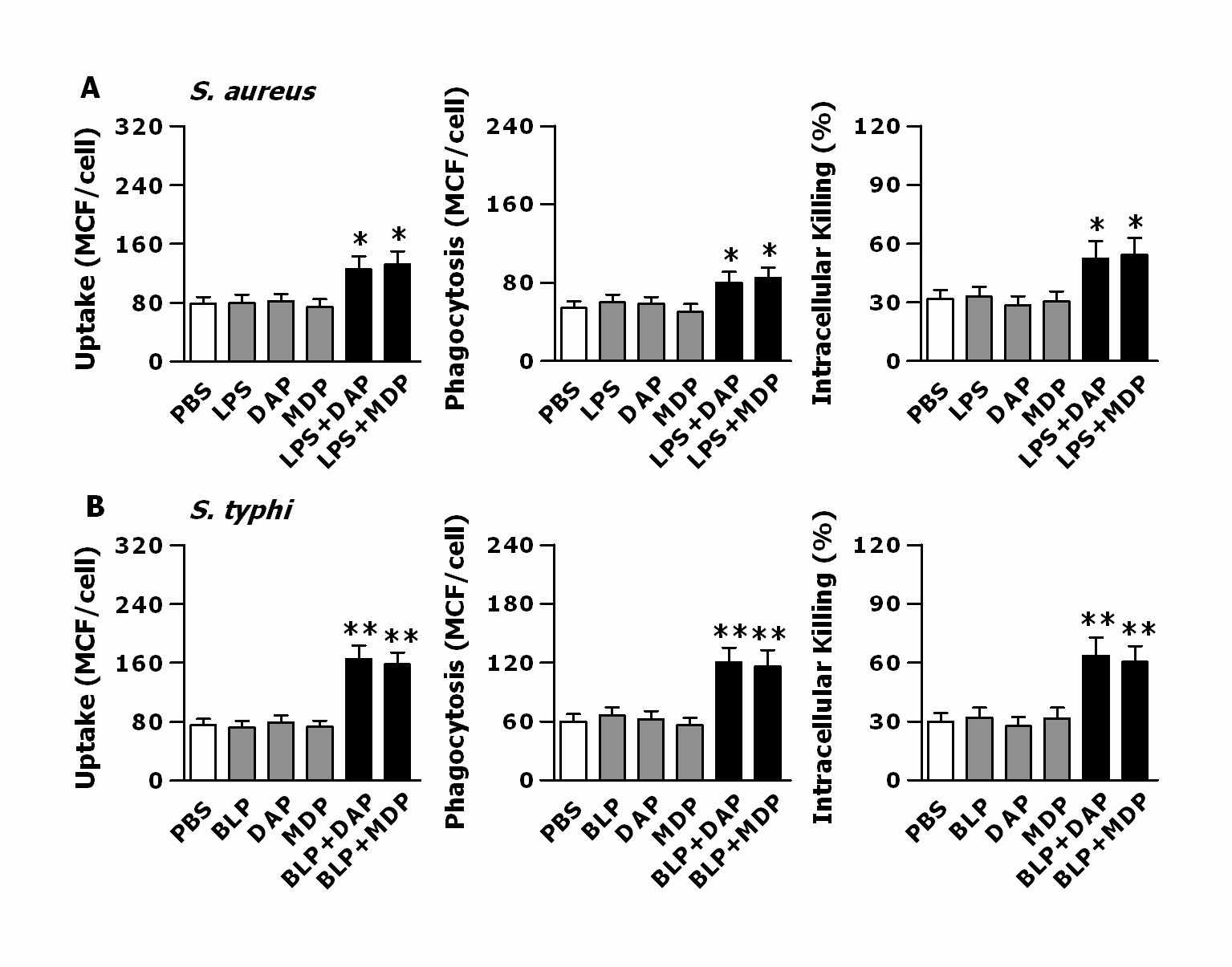


**Figure S2**. Stimulation of macrophages with both TLR and NOD agonists maximizes the innate phagocyte-associated antimicrobial activity. Peritoneal macrophages isolated from wild-type mice were stimulated with LPS (10 ng/ml), Tri-DAP (5 µg/ml), MDP (5 µg/ml), and their combinations (**A**) or BLP (10 ng/ml), Tri-DAP (5 µg/ml), MDP (5 µg/ml), and their combinations (**B**) for 6 h, and further incubated with either FITC-conjugated *S. aureus* (**A**), FITC-conjugated *S. typhimurium* (*S. typhi*) (**B**) for 30 min to assess bacterial uptake and phagocytosis or live *S. aureus* (**A**), live *S. typhi* (**B**) for 60 min to assess intracellular bacterial killing. Bacterial uptake and phagocytosis were expressed as mean channel fluorescence (MCF) per cell. Data are expressed as mean ± SD from four to five independent experiments in triplicate. **p*<0.05, ***p*<0.01 versus macrophages stimulated with LPS, BLP, Tri-DAP, or MDP alone.

**Figure S3**


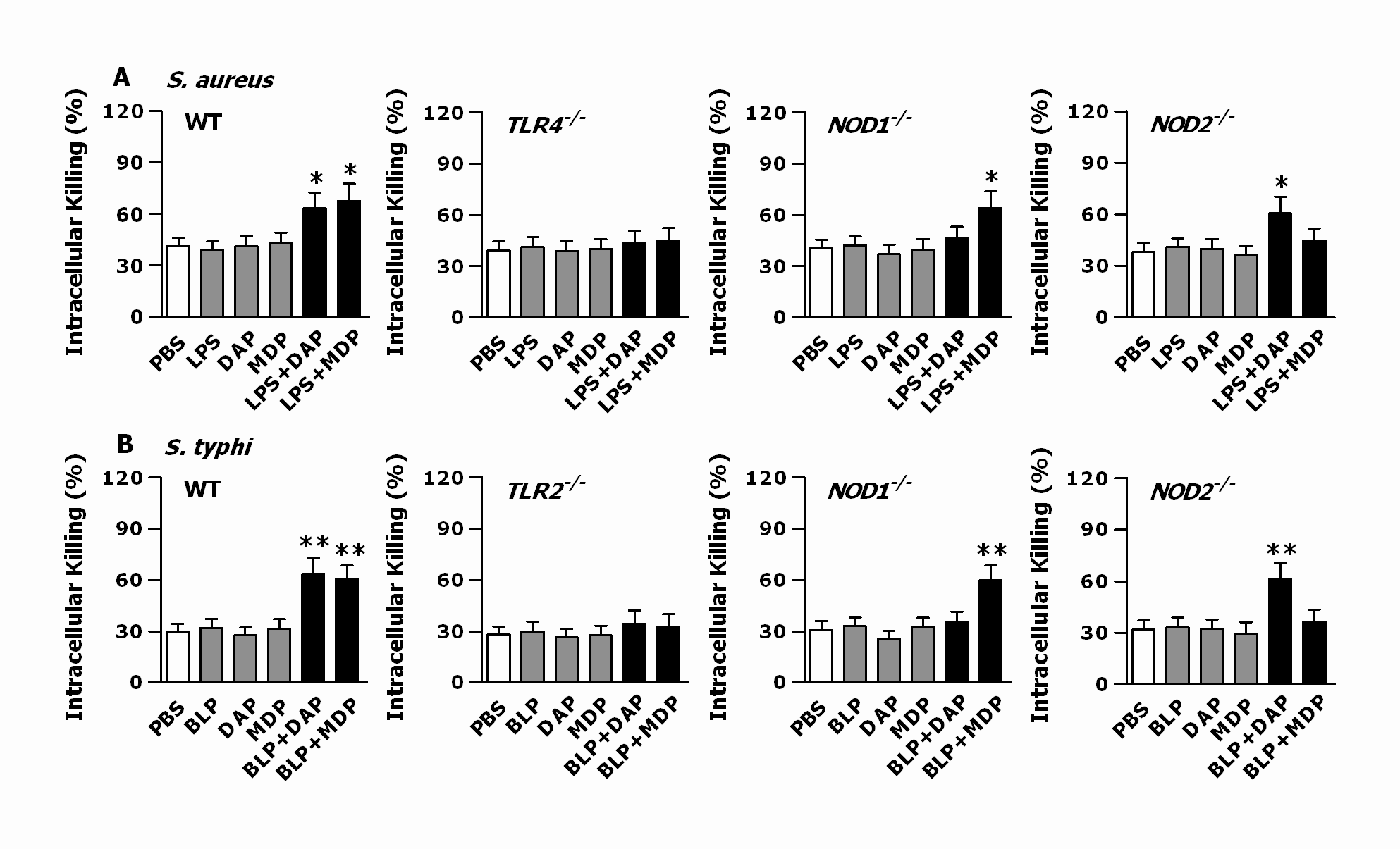


**Figure S3**. TLR and NOD signaling are both required for an efficient macrophage-mediated intracellular bacterial killing. Peritoneal macrophages isolated from wild-type, TLR4- and TLR2-deficient, and NOD1- and NOD2-deficient mice were stimulated with LPS (10 ng/ml), Tri-DAP (5 µg/ml), MDP (5 µg/ml), and their combinations (**A**) or BLP (10 ng/ml), Tri-DAP (5 µg/ml), MDP (5 µg/ml), and their combinations (**B**) for 6 h, and further incubated with live *S. aureus* (**A**) or live *S. typhimurium* (*S. typhi*) (**B**) for 60 min to assess intracellular bacterial killing. Data are expressed as mean ± SD from four to five independent experiments in triplicate. **p*<0.05, ***p*<0.01 versus macrophages stimulated with LPS, BLP, Tri-DAP, or MDP alone.

**Figure S4**


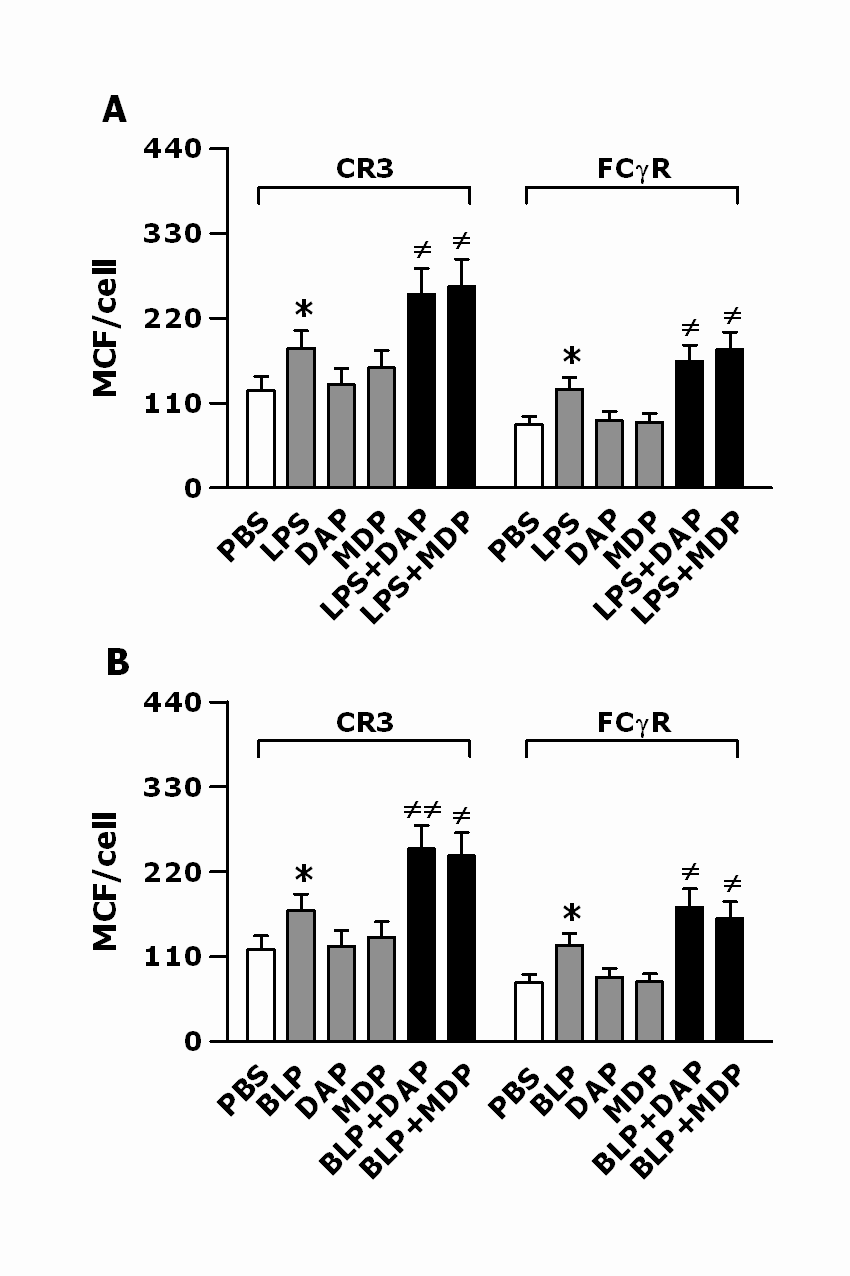


**Figure S4**. LPS or BLP stimulation in combination with Tri-DAP or MDP leads to an enhanced expression of phagocytic receptors on macrophages. Peritoneal macrophages isolated from wild-type mice were stimulated with LPS (10 ng/ml), Tri-DAP (5 µg/ml), MDP (5 µg/ml), and their combinations (**A**) or BLP (10 ng/ml), Tri-DAP (5 µg/ml), MDP (5 µg/ml), and their combinations (**B**) for 2 h. Macrophages incubated with PBS were used as the control. Surface expression of CR3 and FcγR on macrophages was assessed by FACScan analysis and expressed as mean channel fluorescence (MCF) per cell. Data are expressed as mean ± SD from five to six independent experiments in duplicate. **p*<0.05 versus macrophages incubated with PBS, ^≠^*p*<0.05, ^≠≠^*p*<0.01 versus macrophages stimulated with LPS or BLP alone.

**Figure S5**


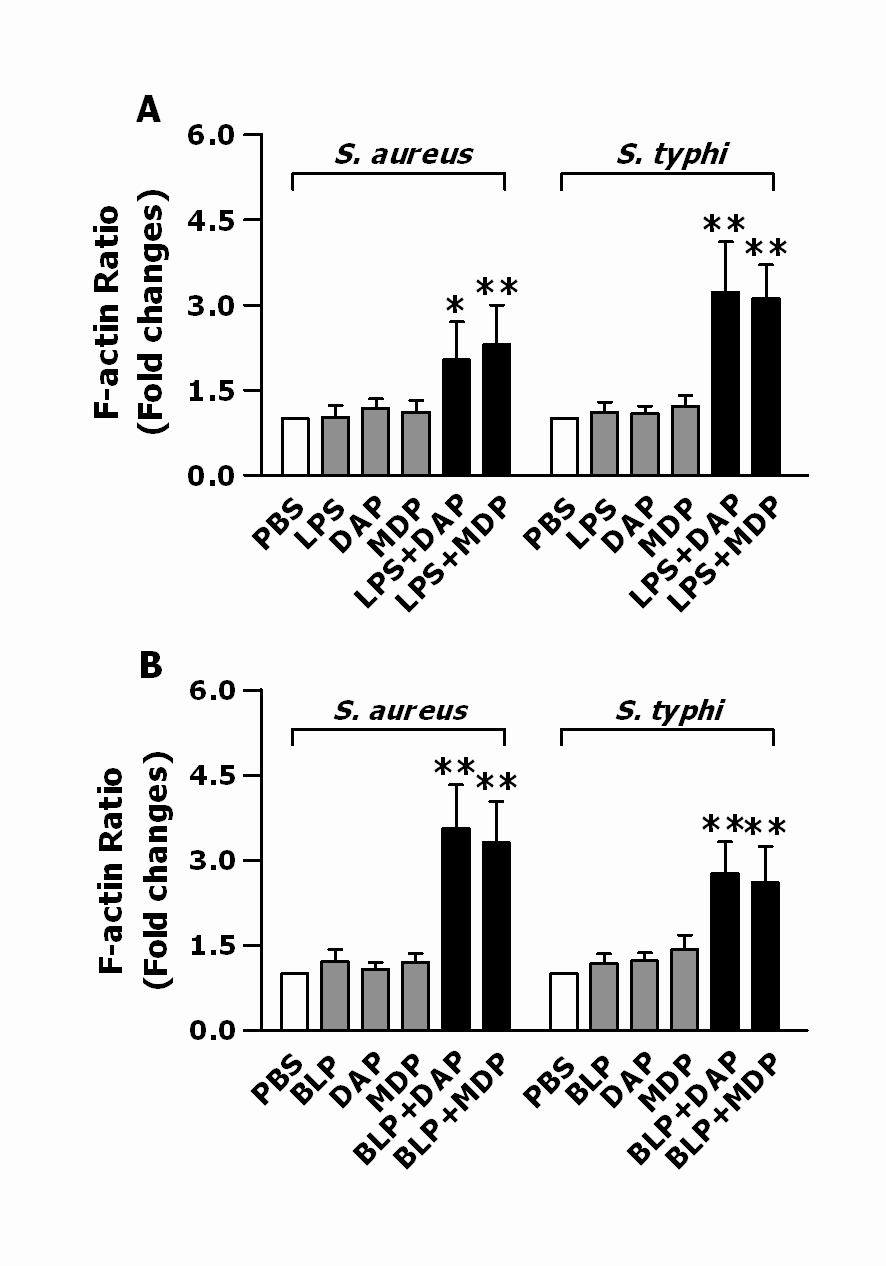


**Figure S5**. Co-stimulation of macrophages with agonists of TLR2/4 and NOD1/2 promotes actin polymerization upon bacteria infection. Peritoneal macrophages isolated from wild-type mice were stimulated with LPS (10 ng/ml), Tri-DAP (5 µg/ml), MDP (5 µg/ml), and their combinations (**A**) or BLP (10 ng/ml), Tri-DAP (5 µg/ml), MDP (5 µg/ml), and their combinations (**B**) for 6 h, and further incubated with heat-killed *S. aureus* (**A**) or *S. typhimurium* (*S. typhi*) (**B**) for 15 min. Macrophages were then fixed, permeabilised, and stained with NBD-phallacidin for extraction of F-actin-bound NBD-phallacidin. Actin polymerization as represented by F-actin contents was assessed by measuring the fluorescent intensity of NBD-phallacidin using a spectrofluorometer and expressed as fold changes. Data are expressed as mean ± SD from five to six independent experiments. **p*<0.05, ***p*<0.01 versus macrophages stimulated with LPS, BLP, Tri-DAP, or MDP alone.

**Figure S6**


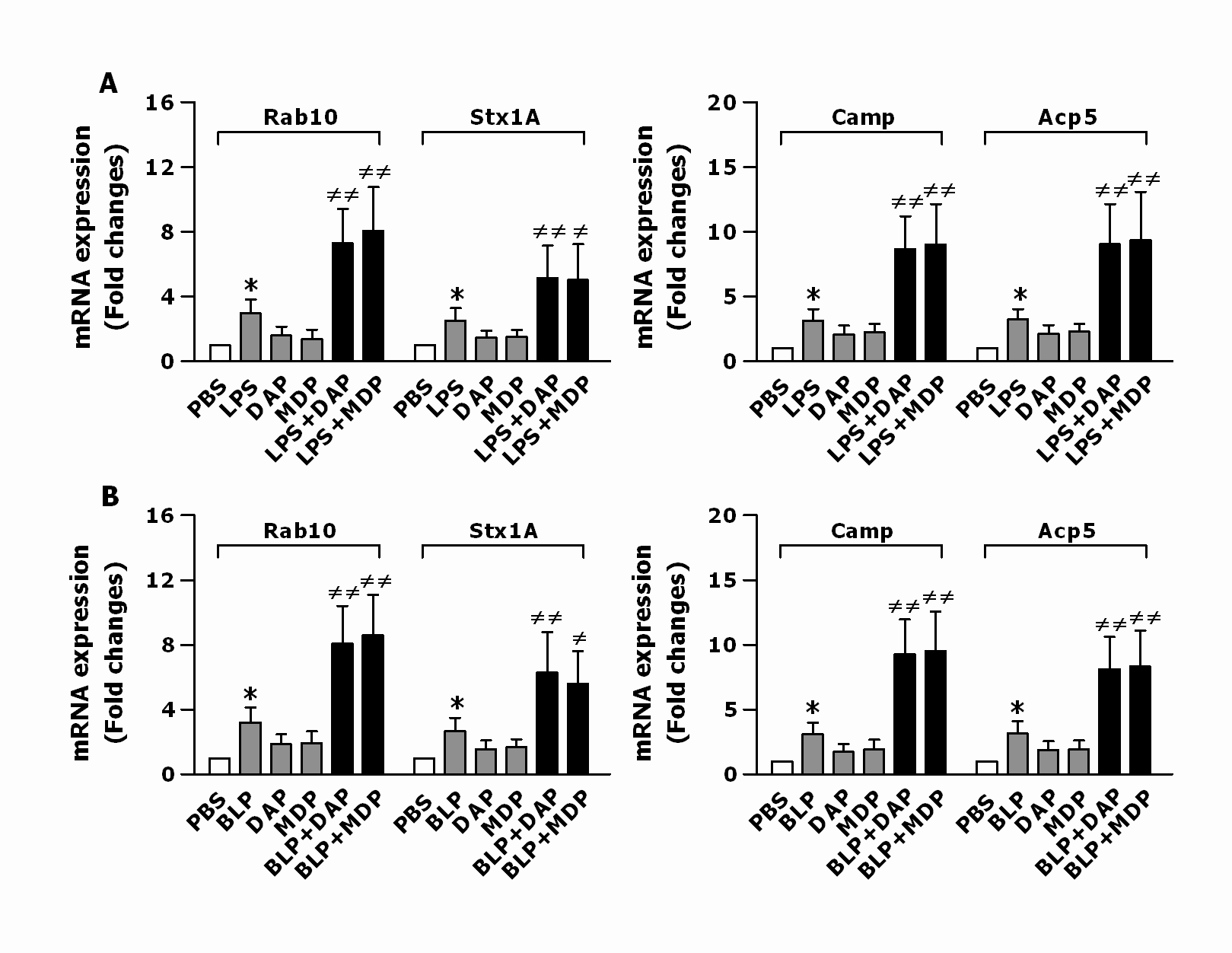


**Figure S6**. LPS or BLP stimulation in combination with Tri-DAP or MDP up-regulates membrane-trafficking regulator and lysosomal enzyme expression. Peritoneal macrophages isolated from wild-type mice were stimulated with LPS (10 ng/ml), Tri-DAP (5 µg/ml), MDP (5 µg/ml), and their combinations (**A**) or BLP (10 ng/ml), Tri-DAP (5 µg/ml), MDP (5 µg/ml), and their combinations (**B**) for 60 min. Macrophages incubated with PBS were used as the control. The mRNA expression of membrane-trafficking regulators Rab10 and Stx1A, and lysosomal enzymes Camp and Acp5 was assessed by quantitative real-time RT-PCR and expressed as fold changes. Data are mean ± SD from at least three independent experiments in duplicate. **p*<0.05 versus macrophages incubated with PBS, ^≠^*p*<0.05, ^≠≠^*p*<0.01 versus macrophages stimulated with LPS or BLP alone.
